# Secondhand Smoke Exposure During Pregnancy and Mothers’ Subsequent Breastfeeding Outcomes: A Systematic Review and Meta-Analysis

**DOI:** 10.1038/s41598-019-44786-z

**Published:** 2019-06-12

**Authors:** Daichi Suzuki, Windy M. V. Wariki, Maiko Suto, Noyuri Yamaji, Yo Takemoto, Mosfequr Rahman, Erika Ota

**Affiliations:** 1 Josai International University, Faculty of Nursing, Department of Nursing, 1 Gumyo, Togane-shi, Chiba, 2838555 Japan; 2 St. Luke’s International University, Graduate School of Nursing Science, Global Health Nursing, 10-1 Akashi-cho, Chuo-ku, Tokyo 1040044 Japan; 3 Sam Ratulangi University, Faculty of Medicine, JL. Kampus UNSRAT, Bahu, Kleak, Malalayang, Kota Manado, Sulawesi Utara 95115 Indonesia; 4 National Center for Child Health and Development, Department of Health Policy, 2-10-1 Okura, Setagaya-ku, Tokyo 1578535 Japan; 5 Juntendo University, Department of Obstetrics and Gynecology, 2-1-1 Hongo, Bunkyo-ku, Tokyo 1130033 Japan; 6 University of Rajshahi, Department of Population Science and Human Resource Development, Rajshahi, 6205 Bangladesh

**Keywords:** Neonatology, Pregnancy outcome

## Abstract

Secondhand smoke exposure of non-smoking women during pregnancy is associated with a higher risk of adverse birth outcomes. However, the available evidence regarding the association between expectant mothers’ secondhand smoke exposure and breastfeeding outcomes remains limited. This systematic review aimed to examine associations between secondhand smoke exposure of nonsmoking women during pregnancy with the initiation, prevalence, and duration or breastfeeding compared to women who were breastfeeding and had not been exposed to secondhand smoke. Women who smoked during pregnancy were excluded. We included case-control, cross-sectional, and cohort studies with a comparison control group. Medline CINAHL, and EMBASE were searched in January 2017. After screening 2777 records we included eight prospective cohort studies. The risk of bias assessment tool for non-randomized studies indicated a high risk of outcome assessment blinding. Meta-analysis of two studies established that the odds of discontinuation of any brestfeeding before six months were significantly increased in the secondhand smoke exposed women (pooled odds = 1.07 [95%CI = 1.01, 1.14], two studies, 1382 women). Therefore, secondhand smoke might be associated with discontinuing any breastfeeding before six months. More research is necessary to understand the association between secondhand smoke and the initiation, prevalence and duration of breastfeeding.

## Introduction

According to the WHO 2016 report, the tobacco smoking epidemic is one of the largest public health problems globally and the number of non-smokers exposed to secondhand smoke (SHS) has been steadily increasing^1^. Smoking during pregnancy is known to induce low birth weight^2,3^, fetal growth retardation^3^, delayed immune development^4^, and reduction in all phases of an infant’s sleep cycle^5^. This is because nicotine diffuses into fetal blood, amniotic fluid, and breast milk and negatively affects neurological development. Therefore, the fetuses and infants of mothers who smoke are at high risk of ill health because of exposure to nicotine^3^. Of additional concern is that maternal SHS exposure is also associated with adverse birth outcomes such as low birth weight^2,6–11^, stillbirth^12^, preterm birth^8,11,13^, spontaneous abortion^12,13^, and birth defects^13^.

Although it is widely known that breastfeeding for the first six months reduces the risk for some adverse events for the infant, the available evidence is limited for the association between SHS and the impact on breastfeeding initiation, prevalence and duration. A study in the US reported that pregnant women who were exposed to SHS had a significantly shorter time (24.9 weeks) of any breastfeeding duration compared to unexposed pregnant women (29.9 weeks). However, there was no significant association with exclusive breastfeeding duration (2.7 weeks for unexposed vs. 2.1 weeks for exposed)^14^. The purpose of this systematic review to examine the associations between secondhand smoke exposure of nonsmoking women during pregnancy with initiation, prevalence, and duration.

## Methods

### Search strategy and selection criteria

The electric database of MEDLINE via Ovid SP and PubMed, CINAHL, and EMBASEwere used to searching as these keywords “secondhand smoke”, “pregnant women”, “case-control”, “cohort”, and “cross-sectional” on January 29, 2017 without any limitations for language and time, or publication status. We collected keywords from literature review, experts’ opinion, and controlled vocabulary (Medical Subject Headings = MeSH, Excerpta Medica Tree = EMTREE, and CINHAL Headings). A medical information specialist developed the search strategy and reported in Appendix 1. The search results were de-duplicated. Two researchers independently screened titles and abstracts.

We defined SHS exposure as contact with SHS from smokers at homes, work places, and other public places. Exclusion criteria included studies of the following: mothers who previously smoked and stopped prior to their pregnancy, pregnant women who smoked during pregnancy, and non-comparative studies. We assessed the association between SHS exposure for women during pregnancy and initiation of breastfeeding, exclusive breastfeeding duration/prevalence or rate, and any or partial breastfeeding duration/prevalence.

### Quality assessment and data extraction

The first author (DS) assigned three dyads (DS & NY; DS & YT and DS & MS) to screen all articles. Next, five dyads, (initial three plus DS & WW and DS & MR) independently performed quality assessment and data extraction. We used the *risk of bias assessment tool for non-randomized studies* (RoBANS)^15^ to assess the risk of bias. Where extracted data and the quality assessment resulted in a discrepancy, the reviewing authors discussed or consulted with all the authors (D.S., W.W., M.S., N.Y., Y.T., M.R., and E.O.) in the team to reach a consensus.

### Data synthesis and analysis

A meta-analysis was performed on studies with similar outcomes. We assessed the effects of secondhand smoke exposure from active smokers. In assessing outcomes an odds ratio (*OR*) was used for dichotomous data, and a student’s t distribution with weighted mean difference (*WMD*) or standardized mean difference (*SMD*) was used for continuous data. The results were presented as means and standard deviations with 95% confidence intervals (*CI*). Data were analyzed using Review Manager (RevMan 5.3.5). Probability (*p)* values of less than 0.05 indicated statistical significance.

Moreover, exclusive breastfeeding is often measured up to six months because complementary feeding starts around same time. Therefore, we set the outcome measurement point up to six months.

## Results

### Description of studies

**Figure 1:**
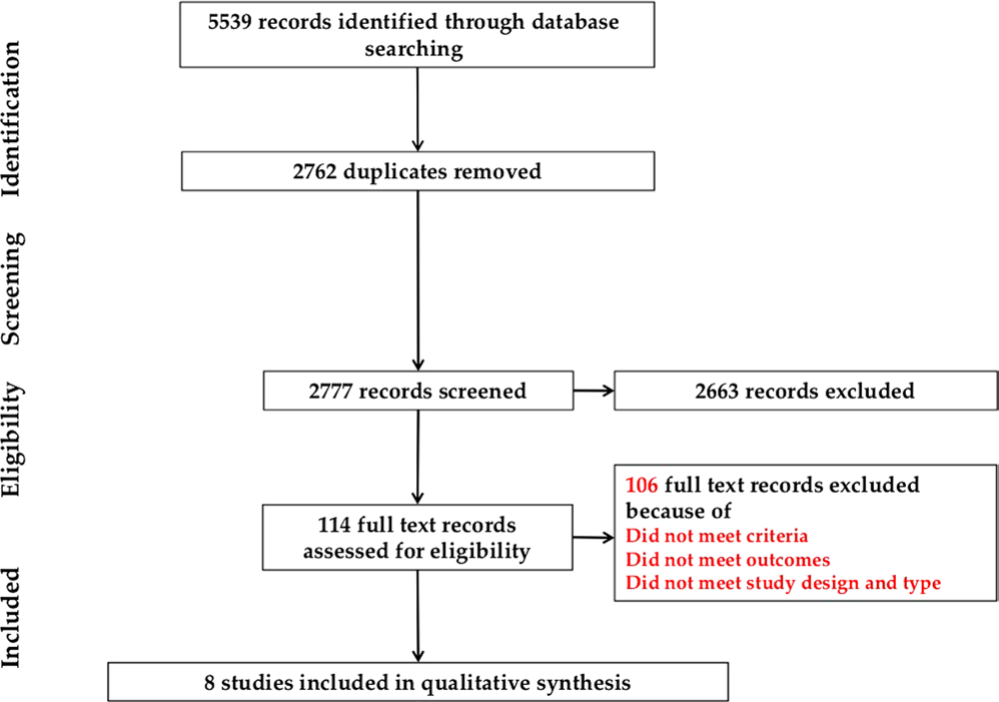
Process of selection of studies has been shown in PRISMA flow diagram.

Our database search identified a total of 5539 records of which 2762 were duplicates and removed. Of the 2777 records screened, 2663 were excluded during the screening because they were irrelevant to our research question. Using the inclusion and exclusion criteria, we selected 114 full texts for assessment. All studies involving smoking cessation in either parent, control participants composed of women who were not exposed to SHS, and pregnant women who smoked during pregnancy, as well as non-comparative studies were then excluded. A total of 106 studies were excluded because of different populations, exposures, outcomes, and study designs. As a result, eight studies were included in the review for data extraction. The selection process of studies is shown in the Preferred Reporting Items Systematic Review Meta-analysis (PRISMA) flow diagram (Fig. 1).

The characteristics of the included studies are shown in Table 1. The studies were published between 1997 and 2014. Two studies were conducted in Poland, and the other six studies were carried out in the United States, Brazil, Iran, Egypt, Taiwan, and Hong Kong^16–23^.

**Table 1:** Characteristics of the included studies.

#	Trial name Year	Country	Setting	Characteristics of Participants			Study Design	Exposure place	Outcome measurement tools	Outcome measurement time	Outcome Assessed
				SHS Exposure	Non SHS exposure	Mean age of Participants Mean ± SD					
1	Baheiraei^23^	Tehran, Iran	5 centers of neonatal thyroid screening within 3–5 days after delivery	170	170	Exposed 27.43 ± 4.34 Non-Exposed 26.56 ± 4.06	Prospective cohort study	Home	self-report and telephone interview	6 months	Exclusive breastfeeding duration and prevalence
2	Chou^19^	Central Taiwan	3 hospitals and community health centers	262	290	—*	Prospective cohort study	Home	interview	6 months	Any or partial breastfeeding rate
3	Horta^21^	Pelotas, Brazil	5 urban maternity hospitals	496	554	—*	Prospective cohort study	Home	structured interview	6 months	Any or partial breastfeeding rate
4	Jedrychowski^17^	Krakow, Poland	Ambulatory prenatal clinics and healthy pregnant women in 1st-2nd trimester of pregnancy delivered birth	332	109	Non-Exposed 27.74 ± 3.48 Low Exposure 28.11 ± 3.31 High Exposure 26.68 ± 3.75	Prospective cohort study	Home	Self-report and Interview	6 months	Breastfeeding duration
5	Kwok^18^	Hong Kong	All 49 governmental maternal and child health centers	4839	1951	—***	Prospective cohort study	Home	self-administered	3 months	Prevalence of breastfeeding
6	Lemke^20^	Tenneessee, USA	Tennessee Children’s Respiratory Initiative	257	194	Exposed 24 [21, 28] ** Non-Exposed 28 [24, 32]**	Prospective cohort study	Home	Self-report and structured Interview	—	Any or partial breastfeeding rate
7	Salama^16^	Assiut city, Egypt	Maternal Health Center of Assiut University Hospital	100	100	Exposed 22.56 ± 3.9 Non-Exposed 22.83 ± 3.5	Prospective cohort study	—***	Questionnaire by the help of well-trained nurse	1 month	Any or partial breastfeeding rate
8	Wdowiak^22^	Lublin, Poland	Clinic for Obstertrics and Gynaecology at the Medical University in Lublin	20	130	—*	Prospective cohort study	Work place	—*	After delivery	Initiation of breastfeeding

*Data was shown by category. Mean age not described.

**Median [IQR].

***Not described.

All studies were prospective cohort studies. The outcomes were: initiation of breastfeeding and problems with breastfeeding^22^, breastfeeding duration^17^, prevalence of breastfeeding^16,18–21^, and breastfeeding duration and prevalence of breast feeding^23^.

The outcome measurement of breastfeeding duration for four studies was six months after delivery^17,19,21,23^. The other measurement points were: three months in Hong Kong^18^; one month after delivery in Egypt^16^, and after delivery in Poland^22^. The measurement point was not described in the study from the USA^20^.

### Risk of bias assessment

**Figure 2:**
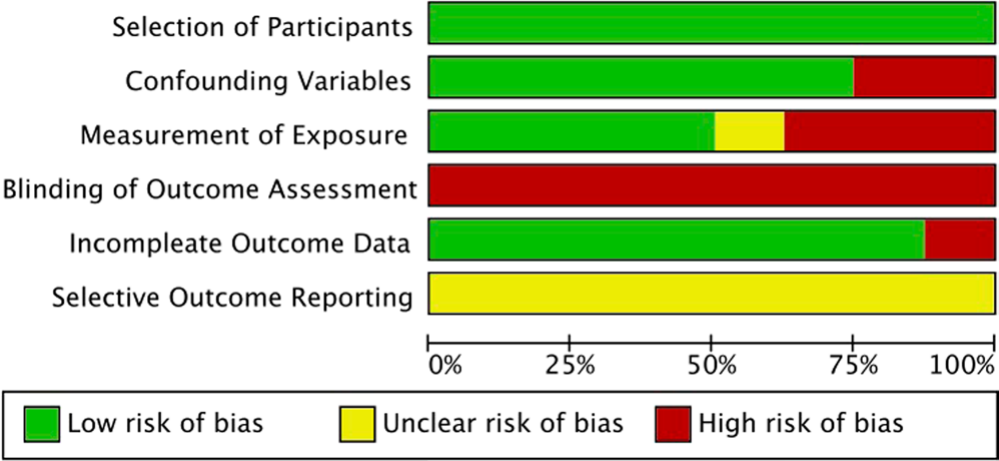
Risk of bias graph review authors’ judgements about each risk of bias item presented as percentages across all included studies.

**Figure 3:**
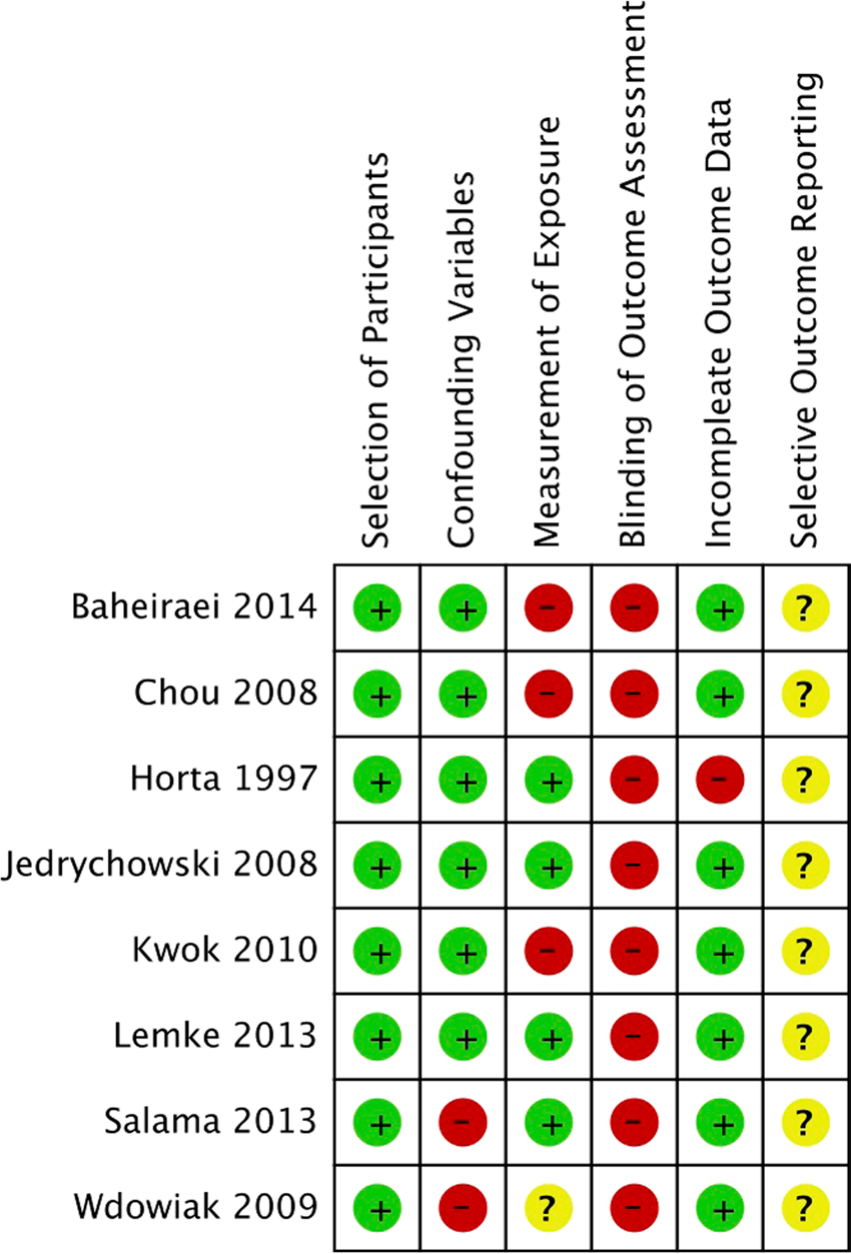
Risk of bias summary review authors’ judgements about each risk of bias item for each included study.

The results of the risk of bias assessment are shown in Figs 2 and 3. There was a high risk of blinding of the outcome assessment because of the reporting bias of self-report measurements (Table 2).

**Table 2:** Judgment of risk of bias assessment.

#	Study	Bias	Author's judgement	Support for judgement
1	Baheiraei^23^	Selection of Participants	Low	Data collected from questionnaire and interview. However, no description about the interviewers. Outcome is self-report measurement.
		Confounding Variables	Low	
		Measurement of Exposure	High	
		Blinding of Outcome Assessment	High	
		Incomplete Outcome Data	Low	
		Selective Outcome Reporting	Unclear	
2	Chou^19^	Selection of Participants	Low	Interviewer were nurses at health center. However, no description about pre-study training. Outcome is self-report measurement.
		Confounding Variables	Low	
		Measurement of Exposure	High	
		Blinding of Outcome Assessment	High	
		Incomplete Outcome Data	Low	
		Selective Outcome Reporting	Unclear	
3	Horta^21^	Selection of Participants	Low	Outcome is self-report measurement. There were some missing data. However, not described.
		Confounding Variables	Low	
		Measurement of Exposure	Low	
		Blinding of Outcome Assessment	High	
		Incomplete Outcome Data	High	
		Selective Outcome Reporting	Unclear	
4	Jedrychowski^17^	Selection of Participants	Low	Outcome is self-report measurement.
		Confounding Variables	Low	
		Measurement of Exposure	Low	
		Blinding of Outcome Assessment	High	
		Incomplete Outcome Data	Low	
		Selective Outcome Reporting	Unclear	
5	Kwok^18^	Selection of Participants	Low	Used the self-administered questionnaire. Outcome is self-report measurement.
		Confounding Variables	Low	
		Measurement of Exposure	High	
		Blinding of Outcome Assessment	High	
		Incomplete Outcome Data	Low	
		Selective Outcome Reporting	Unclear	
6	Lemke^20^	Selection of Participants	Low	Outcome is self-report measurement.
		Confounding Variables	Low	
		Measurement of Exposure	Low	
		Blinding of Outcome Assessment	High	
		Incomplete Outcome Data	Low	
		Selective Outcome Reporting	Unclear	
7	Salama^16^	Selection of Participants	Low	Major variables were confirmed. However, not in analysis phase. Outcome is self-report measurement.
		Confounding Variables	High	
		Measurement of Exposure	Low	
		Blinding of Outcome Assessment	High	
		Incomplete Outcome Data	Low	
		Selective Outcome Reporting	Unclear	
8	Wdowiak^22^	Selection of Participants	Low	Major variables were confirmed. However, not in analysis phase. The Apgar scale used to assess the baby conditions. However, no details of outcome measure were described. Outcome is self-report measurement.
		Confounding Variables	High	
		Measurement of Exposure	Unclear	
		Blinding of Outcome Assessment	High	
		Incomplete Outcome Data	Low	
		Selective Outcome Reporting	Unclear	

### Synthesized meta-analysis and findings

**Figure 4:**
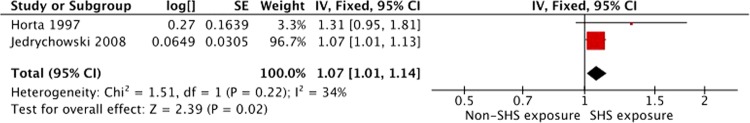
Impact of discontinuation of breastfeeding at 6 months.

Four of the eight studies^16,17,21,23^ assessed the association between SHS exposure and discontinuation of breastfeeding. However, only two studies^17,21^ could be synthesized to indicate the association between SHS and discontinuation of any breastfeeding before six months with 95% CIs. Also, in these two studies, SHS exposure was defined as exposure by any household member. The result of our meta-analysis (Fig. 4) showed there was a significant increased risk of discontinuation of any breastfeeding before six months of those who were exposed to SHS during pregnancy (pooled ORs = 1.07 [95%CI: 1.01–1.14]; *p* = 0.02; *I*^2^ = 34%, 1382 women, 2 studies). Moreover, the results of meta-analysis (Fig. 4) shown that one study was weighted mostly (96%) due to the imprecision. The other two studies^16,23^ also reported breastfeeding duration. However, the study in Iran^23^ only showed the average breastfeeding duration by day. The study in Egypt^16^ measured three outcomes of breastfeeding at one month after delivery. Those two studies used different measurement points and assessments of the outcomes. Therefore, they were excluded from the meta-analysis and instead described narratively.

Moreover, Jedrychowski *et al*.^17^ analyzed blood cotinine levels of mothers as an outcome measure for breastfeeding duration. The results of multiple regression analysis showed that mothers with higher blood cotinine levels had significantly higher odds of discontinuing any breastfeeding before six months (ORs = 1.08 [95%CI: 0.61–1.90]; *p* > z = 0.79; 0.05–0.15 ng/mL of cotinine) vs. (ORs = 2.42 [95%CI: 1.42–4.14]; *p* > z = 0.00; >0.15 ng/mL of cotinine). However, other studies did not use blood cotinine measures, therefore, we used self-reported data of SHS status in the analysis.

### Exclusive breastfeeding duration

Two of the eight studies assessed the relation of the risk of SHS exposure and shortening of exclusive breastfeeding duration^17,23^. Both studies defined the SHS exposure at home and measurement points were six months after delivery. Also, measurement outcomes were the same average duration of exclusive breastfeeding but the method of measuring outcomes was different. The study in Iran reported that the average exclusive breastfeeding period was 27 days shorter at the end of six months for those who were exposed the SHS during pregnancy compared to those who were not exposed^23^. A study in Poland reported that there was a significant inverse association between higher maternal blood cotinine levels and exclusive breastfeeding duration (*r* = −0.195, *p* < 0.0001 [Mean (SD)] = [26.86 (21.69) weeks])^17^.

### Breastfeeding prevalence or rate

Five studies assessed the association between SHS exposure and breastfeeding prevalence^16,18–20,23^. One study measured the prevalence of exclusive breastfeeding^23^, two studies measured the prevalence of any breastfeeding^19,20^, one study measured prevalence of breastfeeding but did not define the types of breastfeeding and need for supplementation^16^, and one study measured the prevalence of partial breastfeeding for any length or exclusive breastfeeding up to three months^18^. There were two studies^19,23^ that measured the prevalence of breastfeeding for the first six months by SHS exposure and non-SHS exposure. Those two studies found that the prevalence of breastfeeding was lower among women who were exposed to SHS during pregnancy. The study in Iran revealed that 22.8% of mothers with SHS exposure breastfed for six months compared to 50.7% with non-SHS exposure^23^. A study in Taiwan^19^ measured the prevalence of breastfeeding for the first six months by comparing SHS exposure versus non-SHS at home and the work place. The results indicated that breastfeeding duration of the SHS exposure group was also shorter compared to the non-SHS group. Those exposed to SHS during the one to five months postpartum period at home had a significantly shorter prevalence of breastfeeding compared to those who were not exposed to SHS. However, there was no significant difference between SHS exposure and prevalence of breastfeeding at six months (AOR = 1.11, [95% CI = 0.44–2.80], 552 women)^19^. In addition, the study in the United States found that breastfeeding among the SHS exposure group was lower compared to the non-SHS exposure group (47% vs. 65%, p < 0.001)^21^. However this study did not describe the measurement point of the outcomes^20^. The study in Egypt compared the associations between SHS exposure and non-SHS exposure on: breastfeeding continuation, supplementation need, and breastfeeding discontinuation^17^. For the SHS exposure group there was a significant decrease in the prevalence of breastfeeding continuation and an increase in the prevalence of supplementation need and breastfeeding discontinuation (SHS exposure vs. non-SHS exposure = 67% vs. 79%; 24% vs. 20%; 9% vs.1%; *p* = 0.008)^16^. The study in Hong Kong^19^ assessed the SHS exposure before or during the early postnatal period by seven SHS exposure situations: prior to pregnancy or not exposed, at postnatal or during pregnancy to the postnatal period, and daily or occasionally. However, they did not describe the exact time of exposure. For occasional exposure 5.2%, and for daily exposure 3.0%. The percentage of partially breastfed for any length of time or exclusively breastfed for less than three months was: 37.7% never exposed, 41.5% occasional, and 44.9% daily exposure^18^.

### Initiation of breastfeeding

The study in Poland^22^ identified the relation of SHS exposure and the occurrence of lactation. This study compared SHS exposed versus non-SHS exposed women during pregnancy and measured the occurrence of lactation by three categories: lactation occurred after delivery, low milk supply, and lactation did not occur. However, there was no significant difference between SHS exposure and initiation of breastfeeding (exposed mother vs non-exposed mother; lactation occurred = 80% vs 88.5%; low milk supply = 15% vs 10%; no lactation occurred = 5% vs 1.5%; *p* = 0.45, 20 vs 130 women).

## Discussion

To our knowledge, this is the first systematic review to assess the association between SHS and breastfeeding. Maternal SHS exposure during pregnancy was significantly associated with an increased discontinuation of any breastfeeding before six months. Also, SHS exposure during pregnancy was associated with shorter breastfeeding duration, and a lower prevalence of breastfeeding. However, we did not synthesize the association of SHS and breastfeeding duration and initiation of breastfeeding and there was no significant association between SHS and initiation of breastfeeding.

Smoking or parenteral nicotine is already known to be associated with low prolactin concentrations^24^. Prolactin is important for metabolic homeostasis and is associated with the lactating mammary gland, by increasing milk proteins, lactose, and lipids^25^. It is important to note that one animal study reported that nicotine was one of risk factors for the inhibition of prolactin release^26^. In addition, tobacco smoke includes more than 4,500 chemicals that are already known to be harmful for humans^27^. SHS actually poses a higher risk than mainstream smoke because SHS is a combination of mainstream smoke, that which is exhaled by the smoker and side stream smoke, that smoke which is emitted from the cigarette between puffs. The side stream smoke includes a higher number of chemicals than mainstream smoke^28^. Therefore, exposure to SHS includes the inhalation of the nicotine or other chemicals, which also affects the smoker. In consideration of these mechanisms and our findings, we surmise that during second hand smoke exposure nicotine or other chemicals are inhaled resulting in the inhibition of prolactin release.

Furthermore, this finding reveals that the duration of breastfeeding among women exposed to second hand smoke tends to be shorter than WHO’s recommendation^29^. WHO/UNICEF recommended infants needed exclusive breastfeeding until six months and then should continue for up to two years old or beyond and complemented with solid food; breastfeeding provides essential nutrition for the infant’s growth and it is an easy way to provide for the protective effects^27^. Breastfeeding can lessen respiratory diseases and diarrhea^29,30^, increase the infant’s growth^30^, and enhance fetal neurodevelopment^29^. Exclusive breastfeeding would be a cost-effective way to protect the infant^31^. Also, the benefit of long-term breastfeeding has already been known to reduce the incidence of obesity/overweight (Pooled OR = 0.74, [95%CI = 0.70–0.78], 105 studies) and type two diabetes (Pooled OR = 0.65, [95%CI = 0.49–0.86], 11 studies)^32^.

Hence, the indications that SHS can affect breastfeeding duration implies that further research is needed to inform public health strategies that could prevent adverse effects of SHS on maternal and child health. Given the benefits of breastfeeding for infant health, an appropriate prevention anti-smoking campaign is critical. In addition, another prevention strategy that protects breastfeeding mothers from SHS is the enactment of a tobacco free law or a tobacco prevention framework like WHO’s MPOWER packages^33^. MPOWER includes six components that protect people from tobacco smoke. MPOWER measures already covered 43% the population globally^34^. Vietnam reported on its smoking ban in public places after they implemented the MPOWER program. They noted that challenges to this complex problem must be addressed on multiple fronts to protect the people from SHS^35^. The government needs to promote precautionary measures to implement public health strategies and advise on the impact of SHS exposure to health^36^.

## Limitation

### Potential mechanisms

Despite our systematic approach to reviewing the extant research on the effects of SHS on breastfeeding there are several limitations that should be addressed. First, only two studies were eligible for meta-analysis. The body of evidence showed low quality and small sample sizes that influenced this review’s quality. Also, outcome measures were of low or very low quality (Table 3). One of the reasons we assessed the quality of evidence as low was because no participants of the studies were blinded to the outcome assessment and it was impossible to have blinded that outcome assessment. There is always the possibility that participants’ reports were untrue if they know the outcome measurement however given the general lack of understanding about the impact of SHS on breastfeeding it is not likely.

**Table 3:** Summary of findings of discontinuing breastfeeding at 6 months.

**Summary of findings:**					
**Effects of Secondhand Smoke on Breastfeeding in Nonsmoking Pregnant Women: A Systematic Review and Meta-Analysis**					
**Patient or population**: Nonsmoking Pregnant Women					
**Setting**: Poland, United States, Brazil, Iran, Egypt, Taiwan, and Hong Kong					
**Intervention**: SHS exposure					
**Comparison**: Non-SHS exposure					
**Outcomes**	Anticipated absolute effects* **(95% CI)**		**Relative effect** **(95% CI)**	**№ of participants** **(studies)**	**Certainty of the evidence** **(GRADE)**
	**Risk with Non-SHS exposure**	**Risk with SHS exposure**			
Discontinuing Breastfeeding at 6 months	0 per 1,000	**0 per 1,000** (0 to 0)	**1.07** (1.01 to 1.14)	(2 observational studies)	⊕⊕◯◯ LOW

*The risk in the intervention group (and its 95% confidence interval) is based on the assumed risk in the comparison group and the **relative effect** of the intervention (and its 95% CI).

**CI:** Confidence interval; **MD:** Mean difference; **OR:** Odds ratio; **HR:** Hazard Ratio.

**GRADE Working Group grades of evidence**.

**High certainty:** We are very confident that the true effect lies close to that of the estimate of the effect.

**Moderate certainty:** We are moderately confident in the effect estimate: The true effect is likely to be close to the estimate of the effect, but there is a possibility that it is substantially different.

**Low certainty:** Our confidence in the effect estimate is limited: The true effect may be substantially different from the estimate of the effect.

**Very low certainty:** We have very little confidence in the effect estimate: The true effect is likely to be substantially different from the estimate of effect.

Second, WHO estimated the prevalence of tobacco smoking of those over 15 years old by each WHO region finding that Europe and the Western Pacific regions were over 20% in 2015^37^. In this review, we included some studies that were conducted in these high smoking prevalence areas and most of the studies that we included were middle to high income countries^38^. Of the two studies eligible for the systematic review and meta-analysis it was found that the higher smoking prevalence was consistently related to lower income levels especially in lower income countries. There was only one study from Africa^39^ where most countries were categorized as lower income countries. Also, Casetta *et al*.^39^ reported there were statistically significant higher ORs of smoking in low-income compared to high-income and middle-income compared to high-income (Pooled OR = 1.69, [95% CI = 1.49–1.92], for low- vs. high-income) vs. (Pooled OR = 1.31, [95% CI = 1.20–1.43], for middle- vs. high-income). In addition, there might be a lack of valid information about smoking data because of the weak data collecting system and infrastructure especially in African and in parts of the Asian region. Therefore, results might change when additional studies are conducted in African and Asian countries categorized as low- or low-middle income countries.

Third, this review included only studies conducted in seven countries: Poland, USA, Brazil, Iran, Egypt, Taiwan, and Hong-Kong. However, according to the WHO there is a higher prevalence of male tobacco smokers in: Indonesia (76.2%), Jordan (70.2%), Kiribati (63.9%), Sierra Leone (60%), and the Russian Federation (59%)^40^ and there were no studies conducted in these countries. By contrast, in some of the countries where there were studies, they had already enacted a tobacco free law or policy, for example Hong-Kong has had a tobacco control office since 2001^41^. The Family Smoking Prevention and Tobacco control act in USA began in 2009 and various states enacted public smoking restrictions from the mid-1980s^42^. Therefore, if studies were conducted in those countries with a high prevalence of male smokers, it might reveal an additional number of pregnant women exposed to SHS and yield different results.

Finally, there are also, some socio-economics indicators, other than SHS, related to longer breastfeeding duration such as mothers who were older, well-educated, married, and in a higher-income bracket compared to lower-income^39^. Mothers who had professional and unprofessional supports had extended breastfeeding duration^43^. Therefore, we need to consider socio-economic status and other potential factors such as the availability of professional supports when examining the association of SHS and mothers’ breastfeeding outcomes.

Future research is required to understand the complex social, psychological and physiological processes involved to prevent the adverse effect of SHS on maternal and child health. Additional research may reveal how SHS exposure is more dangerous for maternal-child health. It will also provide policy arguments for the implementation of public strategies such as smoke free laws. Smoking cessation programs for fathers is one of the ways to protect women and children from SHS exposure especially if it is an intervention from health care professionals^44^. Therefore, there is a need for the implementation of more educational programs or counseling for fathers before delivery in the hospital or health facilities.

## Conclusion

This was a systematic review and meta-analysis of the effects of SHS for women during pregnancy on breastfeeding outcomes. SHS exposure during pregnancy was associated with discontinuation of any breastfeeding before six months compared to no exposure of SHS during pregnancy. However, of the thousands of research articles found only two had sufficient comparability of methods for synthesis and even then the quality of evidence was low indicating that higher quality research is strongly recommended.

## Supplementary information

Appendix
