# Supplementary material for: Secondhand Smoke Exposure During Pregnancy and Mothers’ Subsequent Breastfeeding Outcomes: A Systematic Review and Meta-Analysis
Source: Sci Rep. 2019 Jun 12;9:8535. doi: 10.1038/s41598-019-44786-z (PMC6562041; doi:10.1038/s41598-019-44786-z)
Supplement: Supplementary file 1 — Appendix [file 41598_2019_44786_MOESM1_ESM.doc]

**Secondhand Smoke Exposure During Pregnancy and Mothers’ Subsequent Breastfeeding Outcomes: A Systematic Review and Meta-Analysis**

Daichi Suzuki^1^, Windy M. V. Wariki ^2^, Maiko Suto^3^, Noyuri Yamaji^4^, Yo Takemoto^5^, Md. Mosfequr Rahman^6^, Erika Ota^7^

**1. Daichi Suzuki**, **RN, PHN, MSN;** Josai International University, Faculty of Nursing, Department of Nursing, 1 Gumyo, Togane-shi, Chiba 2838555, Japan; [dusuzki@jiu.ac.jp](mailto:dusuzki@jiu.ac.jp)

St. Luke’s International University, Graduate School of Nursing Science, Global Health Nursing, 10-1 Akashi-cho, Chuo-ku, Tokyo 1040044, Japan; [16mn011@slcn.ac.jp](mailto:16mn011@slcn.ac.jp)

**2. Windy M. V. Wariki, MD, MSc, PhD, Associate Professor;** Sam Ratulangi University, Faculty of Medicine, JL. Kampus UNSRAT, Bahu, Kleak, Malalayang, Kota Manado, Sulawesi Utara 95115, Indonesia; [wwariki@unsrat.ac.id](mailto:wwariki@unsrat.ac.id)

**3. Maiko Suto, PhD;** National Center for Child Health and Development, Department of Health Policy, 2-10-1 Okura, Setagaya-ku, Tokyo 1578535, Japan;

[maiko.suto@gmail.com](mailto:maiko.suto@gmail.com)

**4. Noyuri Yamaji RN, PHN, MSN;** St. Luke’s International University, Graduate School of Nursing Science, Global Health Nursing, 10-1 Akashi-cho, Chuo-ku, Tokyo 1040044, Japan; [16mn017@slcn.ac.jp](mailto:16mn017@slcn.ac.jp)

**5. Yo Takemoto, MD, PhD;** Juntendo University, Department of Obstetrics and Gynecology, 2-1-1 Hongo, Bunkyo-ku, Tokyo 1130033, Japan;

[yotakemoto0402@gmail.com](mailto:yotakemoto0402@gmail.com)

**6. Md. Mosfequr Rahman, MD, PhD, Associate Professor;** University of Rajshahi, Department of Population Science and Human Resource Development, Rajshahi 6205, Bangladesh; <mailto:mosfeque@ru.ac.bd>

**7. Erika Ota, RNM, PhD, Professor (corresponding author);** St. Luke’s International University, Graduate School of Nursing Science, Global Health Nursing, 10-1 Akashi-cho, Chuo-ku, Tokyo 1040044, Japan; <mailto:ota@slcn.ac.jp>

**Appendix: Search strategy**

**I: Search Date**

January 29, 2017

**II: Resources and Number of Results**

**Table 1** Search resources details and number of results

| **Resource** | **Time Coverage** | **Search Interface** | **# of Hits** |
| --- | --- | --- | --- |
| CINAHL [Excluding MEDLINE Records] | 1937 – Search Date | EBSCOhost | 86 |
| EMBASE [Excluding MEDLINE] | 1974 – 2017 Week 4 | Ovid SP | 603 |
| MEDLINE | 1946 – Search Date | Ovid SP | 2672 |
| PubMed | 1946 – Search Date | PubMed | 2178 |
|  | | | |
| Subtotal | 5539 | | |
| Duplicates | 2762 | | |
| **Total (for Screening)** | **2777** | | |

**III: Search Strategies**

***A. CINAHL***

((MH "Passive Smoking") OR TI ( (Passive* OR "Second Hand" OR Secondhand) N6 (Cigar* OR Smok* OR Tobacco) ) OR AB ( (Passive* OR "Second Hand" OR Secondhand) N6 (Cigar* OR Smok* OR Tobacco) )) AND (( (MH "Fetus+") OR (MH "Perinatal Death") OR (MH "Fetal Development+") OR [MH "Gestational Age"] OR (MH "Maternal Exposure") OR (MH "Mothers+") OR (MH "Pregnancy+") OR (MH "Maternal Outcome") OR (MH "Pregnancy Complications+") ) OR TI ( Embryopath* OR Fetal* OR Fetus* OR Foetus OR Foetal OR Gestation* OR Matern* OR Mother* OR Prenat* OR Perinat* OR Pregnan* OR Abort* OR Miscarr* ) OR AB ( Embryopath* OR Fetal* OR Fetus* OR Foetus OR Foetal OR Gestation* OR Matern* OR Mother* OR Prenat* OR Perinat* OR Pregnan* OR Abort* OR Miscarr* )) AND (( (MH "Gestational Age") OR (MH "Nonexperimental Studies") OR (MH "Case Control Studies") OR (MH "Cross Sectional Studies") OR (MH "Prospective Studies+") OR (MH "Retrospective Design") OR (MH "Odds Ratio") ) OR TI ( (Case N12 (Control OR Comparison)) OR Cohort* OR "Cross-Sectional" OR "Cross Section" OR "Follow-Up" OR (Follow* W1 Up) OR Followup OR Incidence* OR Longitudinal OR Observational OR "Odds Ratio" OR "Odds Ratios" OR Prevalence* OR Prospective* OR "Relative Odds" OR Retrospective* OR "Risk Ratio" OR "Risk Ratios" OR Expos* ) OR AB ( (Case N12 (Control OR Comparison)) OR Cohort* OR "Cross-Sectional" OR "Cross Section" OR "Follow-Up" OR (Follow* W1 Up) OR Followup OR Incidence* OR Longitudinal OR Observational OR "Odds Ratio" OR "Odds Ratios" OR Prevalence* OR Prospective* OR "Relative Odds" OR Retrospective* OR "Risk Ratio" OR "Risk Ratios" OR Expos* )) Limiters - Exclude MEDLINE records

***B. EMBASE***

1. Passive Smoking/ OR ((Passive$ OR Second Hand OR Secondhand) adj6 (Cigar$ OR Smok$ OR Tobacco)).ti,ab.
2. Exp Fetus/ OR Exp Fetus Death/ OR Exp Fetus Development/ OR Fetus Mortality/ OR Gestational Age/ OR Perinatal Death/ OR Maternal Exposure/ OR Exp Mother/ OR Exp Pregnancy/ OR Pregnant Woman/ OR Exp Pregnancy Complication/ OR Pregnancy Outcome/ OR Exp Pregnancy Disorder/ OR (Embryopath$ OR F?etal$ OR F?etus$ OR Gestation$ OR Matern$ OR Mother$ OR Prenat$ OR Perinat$ OR Pregnan$ OR Abort$ OR Miscarr$).ti,ab.
3. Exp Case Control Study/ OR Cohort Analysis/ OR Follow Up/ OR Cross-Sectional Study/ OR Prevalence/ OR Observational Study/ OR Odds Ratio/ OR Incidence/ OR Longitudinal Study/ OR ((Case adj12 (Control OR Comparison)) OR Cohort$ OR Cross Section$ OR Follow$ Up OR Followup OR Incidence? OR Longitudinal OR Observational OR Odds Ratio? OR Prevalence? OR Prospective$ OR Relative Odds OR Retrospective$ OR Risk Ratio? OR Expos$).ti,ab.
4. 1 AND 2 AND 3
5. Exp Animals/ OR Exp Invertebrate/ OR Animal Experiment/ OR Animal Model/ OR Animal Tissue/ OR Animal Cell/ OR Nonhuman/
6. Human/ OR Normal Human/ OR Human Cell/
7. 5 AND 6
8. 5 NOT 7
9. 4 NOT 8
10. Limit 9 to Medline
11. 9 NOT 10

***C. MEDLINE***

1. Exp Tobacco Smoke Pollution/ OR ((Passive$ OR Second Hand OR Secondhand) adj6 (Cigar$ OR Smok$ OR Tobacco)).ti,ab.
2. Exp Fetus/ OR Exp Fetal Death/ OR Exp Fetal Development/ OR Exp Fetal Mortality/ OR Exp Gestational Age/ OR Perinatal Death/ OR Exp Maternal Exposure/ OR Exp Mothers/ OR Exp Pregnancy/ OR Exp Pregnancy Complications/ OR Exp Pregnancy Outcome/ OR Exp Pregnant Women/ OR Exp Abortion, Spontaneous/ OR (Embryopath$ OR F?etal$ OR F?etus$ OR Gestation$ OR Matern$ OR Mother$ OR Prenat$ OR Perinat$ OR Pregnan$ OR Abort$ OR Miscarr$).ti,ab.
3. Case-Control Studies/ OR Exp Cohort Studies/ OR Exp Cross-Sectional Studies/ OR Exp Observational Studies as Topic/ OR Exp Odds Ratio/ OR Observational Study.pt. OR ((Case adj12 (Control OR Comparison)) OR Cohort$ OR Cross Section$ OR Follow$ Up OR Followup OR Incidence? OR Longitudinal OR Observational OR Odds Ratio? OR Prevalence? OR Prospective$ OR Relative Odds OR Retrospective$ OR Risk Ratio? OR Expos$).ti,ab. NOT (Animals NOT (Humans and Animals)).sh.
4. 1 AND 2 AND 3

***D. PubMed***

("Tobacco Smoke Pollution"[Mesh] OR ((Passive*[tiab] OR "Second Hand"[tiab] OR Secondhand[tiab]) AND (Cigar*[tiab] OR Smok*[tiab] OR Tobacco[tiab]))) AND ("Fetus"[Mesh] OR "Fetal Death"[Mesh] OR "Fetal Development"[Mesh] OR "Fetal Mortality"[Mesh] OR Embryopath*[tiab] OR Fetal*[tiab] OR Fetus*[tiab] OR Foetus[tiab] OR Foetal[tiab] OR "Gestational Age"[Mesh] OR Gestation*[tiab] OR "Perinatal Death"[Mesh] OR "Maternal Exposure"[Mesh] OR Matern*[tiab] OR "Mothers"[Mesh] OR Mother*[tiab] OR Prenat*[tiab] OR Perinat*[tiab] OR "Pregnancy"[Mesh] OR "Pregnancy Complications"[Mesh] OR "Pregnancy Outcome"[Mesh] OR "Pregnant Women"[Mesh] OR Pregnan*[tiab] OR "Abortion, Spontaneous"[Mesh] OR Abort*[tiab] OR Miscarr*[tiab]) AND ("Case-Control Studies"[Mesh] OR "Cohort Studies"[Mesh] OR "Cross-Sectional Studies"[Mesh] OR "Observational Studies as Topic"[Mesh] OR "Observational Study" [Publication Type] OR "Odds Ratio"[Mesh] OR "Case Comparison"[tiab] OR "Case Control"[tiab] OR Cohort*[tiab] OR "Cross Sectional"[tiab] OR "Cross Section"[tiab] OR "Follow Up"[tiab] OR Followup[tiab] OR Incidence*[tiab] OR Longitudinal[tiab] OR Observational[tiab] OR "Odds Ratio"[tiab] OR "Odds Ratios"[tiab] OR Prevalence*[tiab] OR Prospective*[tiab] OR "Relative Odds"[tiab] OR Retrospective*[tiab] OR "Risk Ratio"[tiab] OR "Risk Ratios"[tiab] OR Expos*[tiab]) AND (Humans[MeSH] NOT (Animals[MeSH] NOT (Animals[MeSH] AND Humans[MeSH])))
